# An Editorial on an Institutional Maternal Request Cesarean Section Rate

**DOI:** 10.7759/cureus.82985

**Published:** 2025-04-25

**Authors:** Thomas J Joyce, Yurina Miyamoto, Chanelle Campbell, Danielle Savastano, Khanh Ta, Angela D Kerr

**Affiliations:** 1 Obstetrics and Gynecology, Richmond University Medical Center, Staten Island, USA

**Keywords:** cesarean section on maternal request, fear of childbirth, healthcare quality assurance, indications of cesarean section, shared decision-making

## Abstract

This editorial examines the elevated rate of maternal request cesarean section (MR C/S) at a high-volume academic institution in New York City. Drawing on internal quality assurance data and patient-reported motivations, it explores the psychological and experiential drivers behind MR C/S. Over a three-month period, 72 primary cesarean deliveries were reviewed, of which 22 (30.6%) were maternal requests. The fear of labor and safety were primary motivators for most patients. This highlights the importance of standardized documentation and patient-centered counseling in addressing this growing trend. This editorial contributes to the growing discourse on MR C/S by highlighting real-world patient motivations at a US community medical center, emphasizing the disconnect between national estimates and institutional documentation, and advocating for standardized reporting frameworks to guide patient counseling and policy reform.

## Editorial

Beginning in the 20th century and moving forward to today, the cesarean section rate (C/S R) has risen drastically in the United States and globally. In 1970, the national rate was 4.5%, compared to 32.1% in 2021. Additionally, an increasing number of pregnant patients are electing for cesarean delivery without medical indication [1,2]. This has contributed to the increasing rise in the cesarean section (C/S) rate, which is thought to be multifactorial in nature, with medical, legal, cultural, and other factors contributing to the rise.

A growing entity known as the maternal request cesarean section (MR C/S) has additionally been increasing in popularity [3]. An MR C/S is defined as a cesarean section (C/S) for a singleton fetus in the vertex position, at term, in the absence of obstetric indication, at the request of the patient. It has been estimated by the American College of Obstetricians and Gynecologists (ACOG) that 2.5% of births have been attributed to an MR C/S. However, ACOG further states that data on MR C/S is limited, and further data is needed to elucidate the entity and evaluate the incidence of the procedure in the population [4].

It is easy to underestimate just how often patients ask for a cesarean by choice. But in our experience, it is far more common than what has been documented in the national dialogue. Understanding MR C/S is essential to interpreting national cesarean trends and shaping meaningful patient counseling. Understanding this is important due to the short- and long-term risks associated with cesarean delivery with respect to the mother and child.

When considering the risk of C/S in the short term, intraoperatively, a patient is at risk of anesthesia complications, internal organ injury, possible hysterectomy, blood transfusion, and injury to the fetus/neonate. Postoperative considerations include longer recovery period and longer hospital stay as compared to normal spontaneous vaginal delivery, along with associated postoperative morbidities. Long-term risks following C/S include increased incidence of intra-abdominal adhesions, increased incidence of malplacentation involving placenta accreta spectrum, and placenta previa in subsequent gestations [1]. During C/S, there are also long-term risks that may affect the child as well, such as asthma, obesity, diabetes, and autoimmune conditions [5]. Given these implications, it is important to understand patient motivations for electing an MR C/S and, as providers, how we can support them.

At our urban, high-volume academic medical center in New York City, we conducted an internal review of primary cesarean deliveries. This was a quality assurance initiative conducted via retrospective chart review over three months, analyzing 72 primary cesarean cases. Those included in this cohort were identified as patients seeking a primary cesarean section for a term vertex fetus without an obstetric indication. Repeat and obstetrically indicated cesarean sections were excluded. Of the 72 primary cesarean sections performed, 22 (30.6%) were identified as maternal requests. As this represents a full institutional cohort rather than a sample, statistical inference measures such as confidence intervals were not applied. This rate significantly exceeded national estimates and prompted a deeper examination of patient motivations. A voluntary, anonymous survey was distributed to these individuals to explore the rationale underlying their decision. Patients meeting criteria were surveyed anonymously; the survey was collected and analyzed descriptively. The limitations of our survey include potential nonresponse bias, as only 13 of 22 MR C/S patients completed the voluntary survey. Additionally, formal inclusion or exclusion criteria for survey participation were not applied beyond patient eligibility based on chart review, which may affect the generalizability of findings.

The most cited reasons were the fear of labor pain, as well as concern for maternal/fetal safety. Additional factors included anxiety about vaginal delivery, concerns regarding prolonged labor, prior negative birth experiences, desire for control, and family influence. These responses highlight a consistent theme across the literature: MR C/S is frequently driven by psychological and emotional factors, often rooted in personal or vicarious experiences [6,7].

It is increasingly common for obstetric patients to inquire about MR C/S [7]. Providers must offer evidence-based education and support patient autonomy during shared decision-making [6]. Importantly, these decisions should not be misconstrued as elective requests based on convenience. Rather, they reflect patient agency within a highly medicalized and risk-conscious obstetric environment. Providers must balance the ethical imperative to respect patient autonomy with the responsibility to communicate the risks and benefits of surgical delivery. Fear of medicolegal consequences may also influence counseling and result in more acceptance of an MR C/S as a practice [8]. MR C/S introduces a unique challenge to shared decision-making, especially when patient preferences diverge from a provider's clinical recommendations. While the institutional MR C/S rate was 30.6%, it is important to note that this may be influenced by our center's high-risk obstetric referral patterns, patient demographics, and local cultural or legal factors, including the heightened awareness of litigation risk in New York City.

At our institution, our rate of 30.6% is far above the national average (2.5%). However, this disparity may be attributable to differences in documentation practices rather than true incidence [4]. These discrepancies in documentation are seen globally as well, such as in Australia, where it is estimated to be 17.3%. However, elsewhere in Europe, it is estimated to be from 7% to 22% [3]. The wide range is thought to be due to alterations in documentation practices. Globally, MR C/S rates are additionally difficult to further elucidate secondary to documentation practices [6]. We believe that this underscores the need for the standardized documentation of MR C/S. Without consistent recording, it is challenging to quantify incidence, assess institutional variation, or allocate appropriate resources. Importantly, clarity in this area could enhance transparency, support clinical policy development, and contribute to a more accurate national dialogue.

In terms of congruence with previous literature, fear seems to be a consistent theme in previous studies [6]. Particularly, the fear of labor pain and concern for maternal and fetal safety have been well-documented as reasons for women choosing to elect for an MR C/S [6]. This has also been reflected in our patient encounters. This reinforces fear as a primary motivator in the decision to pursue MR C/S. As mentioned by ACOG, it is the duty of the provider to fully explain to patients the risks associated with cesarean birth to both the mother and the fetus [4].

Future efforts should incorporate standardized documentation to better assess institutional and global trends in MR C/S. As providers, it is our duty and obligation to help mitigate a patient's fears to help the patient make the most well-informed decision when sharing the burden of their care.
